# Pleiotropic Gene *HMGA2* Regulates Myoblast Proliferation and Affects Body Size of Sheep

**DOI:** 10.3390/ani14182721

**Published:** 2024-09-20

**Authors:** Xiukai Cao, Chen Ling, Yongqi Liu, Yifei Gu, Jinlin Huang, Wei Sun

**Affiliations:** 1 Joint International Research Laboratory of Agriculture and Agri-Product Safety of Ministry of Education of China, Yangzhou University, Yangzhou 225009, China; cxkai0909@163.com; 2 College of Animal Science and Technology, Yangzhou University, Yangzhou 225009, China; 3 Jiangsu Key Laboratory of Zoonosis, Yangzhou University, Yangzhou 225009, China

**Keywords:** sheep, *HMGA2*, myoblast proliferation, core promoter, marker-assisted selection

## Abstract

**Simple Summary:**

The *HMGA2* gene has been known to regulate body size or muscle development in various animals, including mice. In this study, we explored the role of *HMGA2* in sheep. We found that *HMGA2* significantly promotes the proliferation of sheep muscle cells. Additionally, a specific genetic variation in the *HMGA2* gene was identified, which is associated with important growth traits in sheep. These findings suggest that *HMGA2* could be a valuable marker for breeding programs aimed at improving meat production in sheep.

**Abstract:**

Uncovering genes associated with muscle growth and body size will benefit the molecular breeding of meat Hu sheep. *HMGA2* has proven to be an important gene in mouse muscle growth and is associated with the body size of various species. However, its roles in sheep are still limited. Using sheep myoblast as a cell model, the overexpression of *HMGA2* significantly promoted sheep myoblast proliferation, while interference with *HMGA2* expression inhibited proliferation, indicated by qPCR, EdU, and CCK-8 assays. Furthermore, the dual-luciferase reporter system indicated that the region NC_056056.1: 154134300-154134882 (-618 to -1200 bp upstream of the *HMGA2* transcription start site) was one of the habitats of the *HMGA2* core promoter, given the observation that this fragment led to a ~3-fold increase in luciferase activity. Interestingly, SNP rs428001129 (NC_056056.1:g.154134315 C>A) was detected in this fragment by Sanger sequencing of the PCR product of pooled DNA from 458 crossbred sheep. This SNP was found to affect the promoter activity and was significantly associated with chest width at birth and two months old, as well as chest depth at two and six months old. The data obtained in this study demonstrated the phenotypic regulatory role of the *HMGA2* gene in sheep production traits and the potential of rs428001129 in marker-assisted selection for sheep breeding in terms of chest width and chest depth.

## 1. Introduction

Hu sheep, a unique Chinese breed known for its high prolificacy, is an excellent maternal breed for meat sheep crossbreeding improvement [1]. Recently, to meet the growing demand for mutton, the Ministry of Agriculture and Rural Affairs of the People’s Republic of China issued a National Sheep Genetic Improvement Plan (2021–2035) and claimed that improving the yield and quality of mutton is a top priority in the breeding of native Chinese sheep breeds. To this end, carrying out Hu sheep breeding for the purpose of meat production holds significant importance for the development of the sheep industry in China.

Muscle growth is a crucial factor affecting meat production. Myoblasts proliferate and differentiate to form myotubes, determining the number and volume of muscle fibers [2,3]. This process involves the programmed expression and regulation of a series of related genes, such as the newly identified marker genes of muscle growth, *Myomaker* and *Myomerger* [4,5,6]. To date, the molecular regulatory network of muscle growth has not been fully illustrated. Therefore, uncovering more genes associated with muscle growth will benefit the molecular breeding of meat Hu sheep.

In the 1990s, scientists reported a transgenic dwarf mouse with normal growth hormone levels, which was caused by the insertion of exogenous DNA into the *HMGA2* gene [7,8,9]. Currently, dwarf phenotypes caused by *HMGA2* gene knockout or natural mutations have been reported in pig, chicken, and rabbit [10,11,12,13]. Notably, the functional loss of this gene not only reduces animal size but also causes coordinated changes in the development of tissues and organs. Studies have shown that the *HMGA2* gene encodes a structural transcription factor that is exclusively expressed during early animal embryonic development [14]. The HMGA2 protein does not have a transcriptional activation ability itself; instead, it binds to AT-rich motifs in the genome, altering chromatin conformation and promoting the recruitment of transcription factors, thereby regulating the expression of key genes [15,16,17,18,19]. For instance, the HMGA2 protein can bind to the *IGF2BP2* gene, recruit nuclear transcription factor NF-κB, and regulate *IGF2BP2* gene transcription, thereby participating in muscle development and fat metabolism in mice [20]. Additionally, genome-wide association studies (GWASs) have shown that *HMGA2* is significantly associated with body size in horses and dogs and with eye muscle area in cattle and sheep [21,22,23,24,25]. Excitingly, the rs29016809 locus explains ~2% of the body weight and ~4% of the height variation in cattle [26,27]. Therefore, *HMGA2* is not only a major gene regulating body size but also a key gene for muscle development.

At present, direct evidence for the regulatory function of the *HMGA2* gene in sheep muscle growth remains scarce. This study elucidates the proliferative function of *HMGA2* in sheep myoblasts, identifies the core promoter of the *HMGA2* gene, and characterizes a SNP within this core promoter associated with sheep’s body size, thereby providing a potential marker for the molecular breeding of sheep.

## 2. Materials and Methods

### 2.1. Ethics Statement

The experimental animals used in this study were approved by the Yangzhou University Laboratory Animal Welfare and Ethics Committee (No. 202103279), and all experiments followed the ethical principles of animal welfare.

### 2.2. Experimental Animals and Isolation of Sheep Myoblasts

After slaughtering a ewe that was two months pregnant, the fetal sheep was collected for the isolation of ovine myoblasts. Sheep myoblasts were isolated from the *Longissimus dorsi* muscle of the fetal sheep by means of collagenase II as previously described [28]. The cells were cultured in a cell incubator at 37 °C with 5% CO_2_, using Dulbecco’s Modified Eagle Medium (DMEM) (Gibco, Carlsbad, CA, USA) containing 20% Fetal Bovine Serum (FBS) (Gibco, Carlsbad, CA, USA) and 1% penicillin–streptomycin.

Jugular vein blood samples were collected from 458 six-month-old crossbred sheep (Hu sheep × Dorper sheep, 134 rams, 324 ewes, provided by Xuzhou Suyang Co., Ltd., Xuzhou, China), and growth data including body height, body length, chest circumference, chest depth, chest width, and cannon circumference were recorded at the age of birth, one month, two months, three months, and six months for the association analysis.

### 2.3. DNA and RNA Extraction and cDNA Synthesis

Genomic DNA was extracted using the phenol–chloroform method and diluted to 20 ng/μL [29]. Total RNA was extracted using the Trizol method and diluted to 100 ng/μL after quality inspection using NanoDrop Microvolume Spectrophotometers (Thermo Scientific, Carlsbad, CA, USA) [30]. A reverse transcription kit (Takara, Kusatsu, Shiga, Japan) was used to remove genomic DNA from the total RNA, with the following program: 42 °C for 2 min, then stored at −80 °C. The genomic DNA-free total RNA was reverse transcribed using the following program: 37 °C for 15 min, then 85 °C for 5 s. The synthesized cDNA was diluted to 10 ng/μL.

### 2.4. Overexpression and Interference of HMGA2 Gene

Based on the sequence of *HMGA2* (accession number: XM_027967479.3), the full-length *HMGA2* coding sequence (CDS, 330 bp) was synthesized and cloned into the multiple cloning sites (*Hind* III and *Xho* I) of the pcDNA3.1(+) vector (Tsingke Biotechnology Co., Ltd.). *HMGA2* siRNAs (Table 1) were designed and synthesized by Genepharma Co., Ltd. (Suzhou, China).

### 2.5. Detection of Sheep Myoblast Proliferation

When the sheep myoblasts grew to 60% confluence, the recombinant vectors of *HMGA2* overexpression (pcDNA3.1-HMGA2, OE), negative control (pcDNA3.1-blank, NC), small interfering RNA (siRNA), or negative control of siRNA (NC) were transfected into cells with a jetPRIME transfection reagent (Polyplus, USA) with at least three replicates for each condition. After 24 h of transfection, qPCR (quantitative polymerase chain reaction), EdU (5-ethynyl-2′-deoxyuridine), and CCK-8 (cell counting kit-8) assays were performed to detect changes in cell proliferation.

qPCR: SYBR Premix Ex Taq II (Takara, Japan) was used for the relative expression of marker genes (*CDK2* and *CCND1*) of cell proliferation [31]. Reaction system: Taq Ⅱ 12.5 μL, 0.4 pmol/μL forward primers (final concentration), 0.4 pmol/μL reverse primers (final concentration), cDNA 2 μL, and ddH_2_O 8.5 μL. Reaction program: 95 °C for 30 s, 40 cycles of 95 °C for 5 s, 60 °C for 30 s, and 95 °C for 10 s. 2^-∆∆Ct^ was used to determine the mRNA expression levels. The primers used for qPCR are listed in Table 1.

EdU: EdU reagent A (50 μM) was diluted with a complete medium at a ratio of 1000:1. When cells grew to 80% confluence, reagent A was added, and the cells were incubated at 37 °C for 2 h. Cells were then fixed with 4% paraformaldehyde at room temperature for 30 min. Glycine (2 mg/mL), a permeabilization agent (0.5% Triton X-100), a 1× Apollo staining reaction solution, and a Hoechst 33342 reaction solution were added for staining according to the instructions (Ribobio, China). A fluorescent inverted microscope was used to detect the staining.

CCK-8: Cell counting was carried out with a CCK-8 kit (Beyotime, Shanghai, China). Each treatment had six parallel wells. For each well, 10 μL of CCK-8 reagent was added and incubated at 37 °C for 2 h. Finally, cell counting was presented as the OD value at 450 nm with a microplate reader.

### 2.6. Determination of HMGA2 Promoter Activity

Three regions were predicted to be core promoters of the *HMGA2* gene using Neural Network Promoter Prediction (https://www.fruitfly.org/seq_tools/promoter.html, accessed on 17 September 2024). The first of them was predicted at NC_056056.1: 154135457- 154135507, upstream of the *HMGA2* transcription start site (TSS) at -1775 to -1825 bp; the second was predicted at NC_056056.1: 154135093- 154135143 (upstream of TSS, -1411 to -1461 bp); the last one was predicted at NC_056056.1: 154134700- 154134750 (upstream of TSS, -1018 to -1068 bp). Based on the locations of the predicted core promoters, primers (Table 1) were designed using Vazyme software (https://crm.vazyme.com/cetool/singlefragment.html, accessed on 17 September 2024). A series of promoter fragments (named P1, P2, P3, and P4) containing different core promoters were amplified by PCR according to the instructions of the Takara PrimeSTAR Max Premix (2×) (Takara, Japan). P1 is 1942 bp and located at NC_056056.1: 154133741-154135682 (upstream of TSS, -59 to -2000 bp); P2 is 1541 bp and located at NC_056056.1: 154133741-154135281 (upstream of TSS, -59 to -1599 bp); P3 is 1142 bp and located at NC_056056.1: 154133741-154134882 (upstream of TSS, -59 to -1200 bp); P4 is 560 bp and located at NC_056056.1: 154133741-154134300 (upstream of TSS, -59 to -618 bp). PCR program: 98 °C for 10 s, 68 °C for 15 s, 72 °C for 1 min, and 39 cycles. The PCR products and pGL3-Basic were digested with *Xho* I and *Nhe* I and then purified and recovered according to the instructions of the SanPrep Column PCR Product Purification Kit (Sangon Biotech, Shanghai, China) and SanPrep Column DNA Gel Extraction Kit (Sangon Biotech, Shanghai, China), respectively. The target fragment was ligated with linearized pGL3-Basic using a DNA Ligation Kit (Takara, Kusatsu, Shiga, Japan). The reaction system included 200 U T4 ligase, 50 ng target fragment, and 50 ng linearized pGL3-Basic, and the reaction was incubated overnight at 16 °C. The ligation product (5.0 μL) was added to 30 μL DH5α and incubated in a 37 °C shaker for 1.5 h. Positive clones were selected by Sanger sequencing, and plasmid extraction was performed using the SanPrep Column Plasmid Mini-Preps Kit (Sangon Biotech, Shanghai, China).

The recombinant pGL3-Basic (coding firefly luciferase) was co-transfected with pRL-TK plasmid (internal control, coding Renilla luciferase) into 293T cells. After 24 h of incubation, the luciferase activities were measured with the Dual-Glo Luciferase Assay System (Promega, USA) according to the kit instructions. A microplate reader was used to qualify the luciferase activities of five replicates of each group, and the firefly luciferase activity was normalized against the Renilla luciferase activity.

### 2.7. Identification and Genotyping of SNP in the HMGA2 Core Promoter

A DNA pool of 50 sheep, each contributing 2.0 μL DNA, was created for SNP identification. The primers SNP-F and SNP-R (Table 1) were used for PCR amplification and SNP scanning. PCR reaction system (25 μL): DNA 20 ng, 0.4 pmol/μL forward primers (final concentration), 0.4 pmol/μL reverse primers (final concentration), PrimeSTAR Max Premix (Takara, Kusatsu, Shiga, Japan) 5.0 μL, and ddH_2_O 2 μL. PCR program: 30 cycles of 98 °C for 10 s, 60 °C for 15 s, and 72 °C for 1 min. PCR products were sequenced by Sanger sequencing (Sangon Biotech, Shanghai, China) to detect variations. The presence of two peaks at a particular base position in a Sanger sequencing chromatogram is indicative of a single nucleotide polymorphism (SNP) at that locus. Given the lack of appropriate restriction enzymes, Sanger sequencing was chosen as the genotyping method for the rs428001129 locus (NC_056056.1:g.154134315 C>A) in each sheep. The same primers and Sanger sequencing were used for genotyping of the 458 sheep as well.

### 2.8. Statistical Analysis

One-way ANOVA and t-test were used for the cell proliferation assay data and dual-luciferase reporter assay data. The genotype frequency, allele frequency, and Hardy–Weinberg equilibrium (HWE) were calculated using SHEsis [32]. The population’s genetic indices such as the observed heterozygosity (He), observed homozygosity (Ho), effective allele numbers (Ne), and polymorphism information content (PIC) were calculated with POPGENE. The association analysis was conducted using the general linear model (GLM) of SPSS21.0, with sex and genotype as fixed effects. Results were presented as Mean ± SE. Results were considered significant at * *p* < 0.05, ** *p* < 0.01, and *** *p* < 0.001.

## 3. Results

### 3.1. Efficiencies of Overexpression and Interference

**Figure 1:**
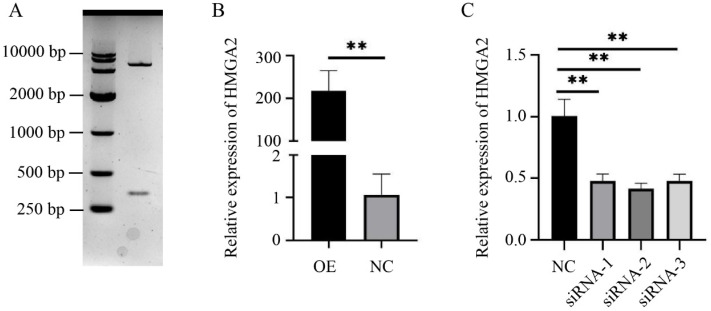
Efficiency detection of *HMGA2* overexpression and interference. (**A**) Agarose gel electrophoresis of the recombinant overexpression vector pcDNA3.1(+)-HMGA2 digested with *Hind* Ⅲ and *Xho* I; (**B**) overexpression efficiency of pcDNA3.1(+)-HMGA2 (OE); (**C**) comparison of interference efficiency of three *HMGA2* siRNAs. ** *p* < 0.01.

The recombinant overexpression vector pcDNA3.1(+)-HMGA2 was digested with *Hind* Ⅲ and *Xho* I, resulting in two bands, with the smaller band being the *HMGA2* CDS 330 bp (Figure 1A). When the pcDNA3.1(+)-HMGA2 vector was transfected into sheep myoblasts, the *HMGA2* expression increased approximately 200-fold (Figure 1B). To screen effective siRNAs, the synthesized *HMGA2* siRNAs were transfected into sheep myoblasts, and siRNA-2 was found to have the best interference efficiency, reducing the *HMGA2* mRNA expression by approximately 60% compared to siRNA-NC (*p* < 0.01) (Figure 1C). Therefore, pcDNA3.1(+)-HMGA2 and siRNA-2 could be used for subsequent experiments.

### 3.2. HMGA2 Promotes Proliferation of Sheep Myoblasts

**Figure 2:**
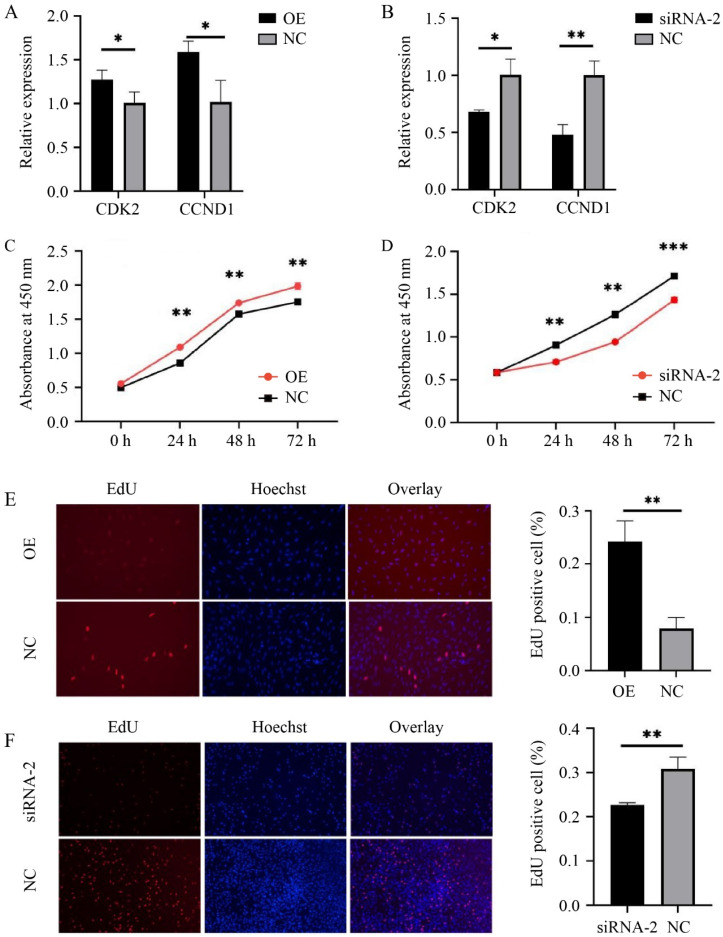
*HMGA2* promotes proliferation of sheep myoblasts. (**A**,**B**) The relative expression of the marker genes of cell proliferation after *HMGA2* overexpression or interference; (**C**,**D**) cell viability, detected by CCK-8 (presented as the OD value at 450 nm); (**E**,**F**) the proportion of EdU-positive cells (blue: live cells, red: proliferating cells). * *p* < 0.05, ** *p* < 0.01, and *** *p* < 0.001.

Compared to the control group (NC), the mRNA levels of the cell proliferation marker genes *CDK2* (*p* < 0.05) and *CCND1* (*p* < 0.05) were significantly increased after *HMGA2* overexpression (Figure 2A). Conversely, the expression levels of *CDK2* (*p* < 0.05) and *CCND1* (*p* < 0.01) were significantly decreased after interfering with *HMGA2* (Figure 2B). The sheep myoblasts were transfected with pcDNA3.1(+)-HMGA2, siRNA-2, and a negative control, respectively, and their cell viability was detected using the CCK-8 assay at 0 h, 24 h, 48 h, and 72 h after transfection (presented as OD value at 450 nm). The results in Figure 2C,D show that the cell viability was significantly higher in the *HMGA2* overexpression group compared to the control group (*p* < 0.01), while the cell viability was significantly lower in the *HMGA2* interference group compared to the control group (*p* < 0.01).

To further determine the effect of the *HMGA2* gene on sheep myoblast proliferation, an EdU assay was carried out. As shown in Figure 2E,F, blue fluorescence represents live cells, and red fluorescence represents proliferating cells, with a higher proportion of red indicating more proliferating cells. ImageJ was used for cell counting, and the proportion of EdU-positive cells (red fluorescence) was calculated. Compared to the control group, the proportion of EdU-positive cells was significantly increased by approximately 2.5-fold after *HMGA2* overexpression (Figure 2E, *p* < 0.01), while it was significantly decreased by approximately 1.5-fold after *HMGA2* interference (Figure 2F, *p* < 0.01). These findings indicate that the *HMGA2* gene can promote myoblast proliferation in sheep, which is consistent with its role in mice.

### 3.3. SNP rs428001129 within HMGA2 Promoter Affects Promoter Activity

**Figure 3:**
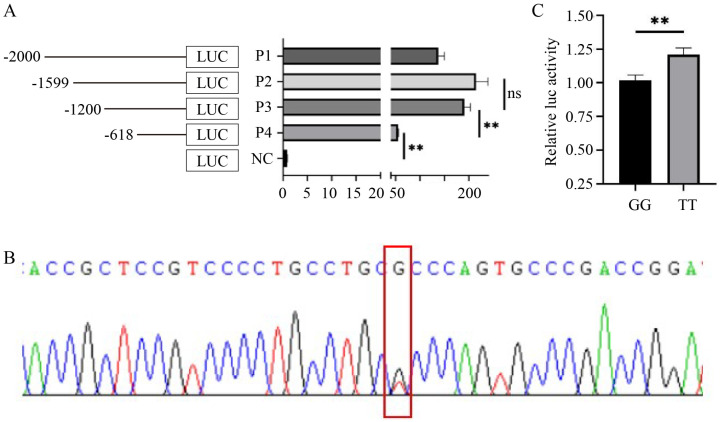
Identification of *HMGA2* core promoter and variation. (**A**) Relative luciferase activities of different lengths of promoter fragments; (**B**) Sanger sequencing detected SNP rs428001129 in NC_056056.1: 154134300-154134882 (-618 to -1200 bp upstream of the *HMGA2* TSS) containing a core promoter; (**C**) rs428001129 changed *HMGA2* promoter activity. P1, NC_056056.1: 154133741-154135682 (upstream of TSS, -59 to -2000 bp); P2, NC_056056.1: 154133741-154135281 (upstream of TSS, -59 to -1599 bp); P3, NC_056056.1: 154133741-154134882 (upstream of TSS, -59 to -1200 bp); P4, NC_056056.1: 154133741-154134300 (upstream of TSS, -59 to -618 bp). ** *p* < 0.01.

Four different lengths of promoter fragments were amplified, named P1, P2, P3, and P4, and cloned into the pGL3-Basic vector (Figure 3A). The luciferase activities of P1, P2, P3, and P4 were significantly higher than those of the NC (blank pGL3-Basic) group (*p* < 0.01). However, the luciferase activity of P3 increased ~3-fold compared to P4, while no significant differences between P2 and P3 were observed (*p* > 0.05), suggesting that the region NC_056056.1: 154134300-154134882 (-618 to -1200 bp upstream of the *HMGA2* TSS) contains a core promoter of the *HMGA2* gene, but we cannot exclude the possibility that the region P4 has additional core promoter(s).

Using pooled DNA as a template, a fragment containing the *HMGA2* core promoter was amplified, followed by Sanger sequencing. An SNP, annotated as rs428001129 (NC_056056.1:g.154134315C>A) in the Ensembl database (ensembl.org), was detected in NC_056056.1: 154134300-154134882 (-618 to -1200 bp upstream of the *HMGA2* TSS) (Figure 3B). The amplified products from the DNA of wild-type (GG) and mutant (TT) individuals using SNP-F/R primers were cloned into the pGL3-Basic vector. As shown in Figure 3C, the TT genotype increased the relative luciferase activity remarkably compared to GG (*p* < 0.01). These results suggest that rs428001129 could change *HMGA2* promoter activity and may be associated with sheep growth traits.

### 3.4. Efficiencies of Overexpression and Interference

Sanger sequencing was used to genotype the SNP rs428001129 in 458 sheep. The genotype frequencies of wild GG, heterozygous GT, and mutant TT were 0.478, 0.454, and 0.068, respectively. The allele frequency of G (0.705) was higher than that of T (0.295). The observed heterozygosity (He) was 0.416, the observed homozygosity (Ho) was 0.584, and the effective allele number (Ne) was 1.712. Notably, rs428001129 was a moderate polymorphism (0.25 < PIC = 0.329 < 0.50) and in Hardy–Weinberg equilibrium, suggesting that rs428001129 does not undergo selection in the crossbred sheep population (Hu sheep × Dorper sheep) used in this study.

The results of the association analysis are shown in Table 2. The SNP rs428001129 was significantly associated with chest width at birth (*p* < 0.01) and two months old (*p* < 0.01) and chest depth at two months old (*p* < 0.05) and six months old (*p* < 0.01). At birth, the chest width of TT individuals was significantly larger than that of GG and GT individuals, with no significant difference between GG and GT. At two months old, TT individuals had significantly larger chest depth than GG; for chest width, individuals with the TT or GT genotype were larger than GG, with no significant differences between TT and GT. At six months old, TT individuals had significantly larger chest depths than GG and GT individuals, with no significant difference between GT and GG. Notably, at all ages, TT individuals tended to have better performance in body weights than GG and GT individuals, but the differences were not significant. These results suggest that rs428001129 can be used for marker-assisted selection (MAS) in sheep, with the TT being the favorable genotype, conferring significantly larger chest width and depth and slightly higher body weight.

## 4. Discussion

The *HMGA2* gene encodes a non-histone chromatin-binding protein that plays a crucial role in the regulation of gene expression and chromatin remodeling. Using the Expression Atlas in the Ruminant Genome Database (http://animal.omics.pro/code/index.php/RGD, accessed on 17 September 2024), we found that the expression profiling of the *HMGA2* gene is consistent across different species. *HMGA2* is highly expressed during early embryonic development, playing a vital role in normal embryogenesis. In adult tissues, the expression of the *HMGA2* gene is very low or absent, but it may be upregulated in certain tissues and under certain conditions. For example, in testis and ovary, it is involved in maintaining and differentiating stem cells [33]. Furthermore, *HMGA2* is often found to be highly expressed in various types of tumors and has garnered significant attention in cancer research, including research on breast cancer, lung cancer, pancreatic cancer, etc. [34]. This overexpression can promote cell proliferation, inhibit apoptosis, and enhance invasive capabilities through different mechanisms, thereby driving tumor development [34].

This study demonstrated that the *HMGA2* gene promotes the proliferation of sheep myoblasts and is associated with body size, mirroring its function in various species, including mice, pigs, chickens, and rabbits [10,11,12,13,20]. These results indicated that in addition to its conserved expression pattern, the *HMGA2* gene appears to have a conserved phenotypic effect. The conserved phenotypic effect of the *HMGA2* gene appears to be related to its sequence. The full length of the sheep *HMGA2* gene is approximately 145 kb, with the length of intron 3 alone reaching about 117 kb, while the coding region is only 330 bp, which is almost identical across mammals. To date, the phenotypic effects of the *HMGA2* gene and their corresponding molecular mechanisms have been extensively studied, including the *LIN28B-let7-HMGA2-IGF2BP2* pathway in muscle development and the *HMGA2-PLAG1-IGF2* pathway in body size [35,36,37,38,39].

In contrast, revealing the *cis*-regulatory elements of the *HMGA2* gene is more likely to capture the interest of researchers and breeders. *Cis*-regulatory elements are specific DNA sequences in or near a gene that are required for the proper spatiotemporal expression of that gene (although the distance of the regulated gene may be many megabases away), often characteristically containing binding sites for transcription factors [40]. Promoters, enhancers, silencers, and insulators are examples of *cis*-regulatory elements. Genetic variations in *cis*-regulatory elements could directly impact the gene expression, making them potential markers for disease diagnostics and molecular breeding. The ENCODE cCREs database (https://screen.encodeproject.org/, accessed on 17 September 2024) annotates a large number of *cis*-regulatory elements within the third intron of the human and mouse *HMGA2* genes. Among these, an enhancer within the human *HMGA2* gene’s third intron mediates TET1-promoted cell growth in human liver cancer [41]. For livestock, the cattle *HMGA2* intronic enhancer (chr5: 47901023–47902297) in a strong sweep region has been confirmed to promote the proliferation of bovine myoblasts [42].

The transcriptional regulation by enhancers depends on their interaction with promoters. Therefore, identifying the core promoter of a gene is beneficial for improving the accuracy of enhancer annotation. To identify the core promoter of the sheep *HMGA2* gene, the pGL3-Basic/pRL-TK dual-luciferase reporter system was utilized [43]. Firefly and Renilla luciferases have high sensitivity and efficiency and are popularly employed as reporter genes, which oxidize different substrates to generate quantifiable luminescence [44]. In this study, a series of promoter deletion fragments of *HMGA2* were cloned into the promoter region of the firefly luciferase gene of the pGL3-Basic vector (Addgene #212936) with the Renilla luciferase of the pRL-TK vector as an internal control. Therefore, the luminescence ratio can reflect the expression level of firefly luciferase, thereby indicating the promoter activity of different deletion fragments. In other words, a higher luminescence ratio corresponds to higher promoter activity of the deletion fragment. Here, by comparing the relative luciferase activities of four promoter deletion fragments of *HMGA2*, the region of NC_056056.1: 154134300-154134882 (-618 bp to -1200 bp upstream of TSS) was putatively inferred as one of the habitats of the *HMGA2* core promoter. However, this study could not exclude the possibility of other regions also containing the core promoter(s) of the *HMGA2* gene, such as the P4 region. This is because further deletion of the P4 fragment was not performed, which is a limitation of this research.

In the fragment of NC_056056.1: 154134300-154134882 (-618 to -1200 bp upstream of the *HMGA2* TSS), SNP rs428001129 was detected. It was significantly associated with chest width and depth and had a detectable but not significant effect on the body weight of the crossbred sheep population (Hu sheep × Dorper sheep). A previous study identified SNP and Indel variations in *HMGA2*, associated with several growth traits in Awassi and Karakul sheep, referred to as ‘intron1:45 A > T’ and ‘intron2:42 A del’ [45]; however, due to the lack of detailed sequence information, we are unable to accurately determine the exact positions of these variations. Together, all these findings provide us with sufficient reason to believe that *HMGA2* is the major gene regulating sheep growth and development. Therefore, rs428001129-influenced *HMGA2* promoter activity can serve as a molecular marker for the breeding of meat sheep. However, due to the lack of embryonic tissue samples, this study did not investigate the impact of rs428001129 on the *HMGA2* gene expression, which is another limitation of this research. In the future, a more in-depth investigation of rs428001129 will be conducted, particularly concerning its potential effects on transcription factor binding.

## 5. Conclusions

The *HMGA2* gene promotes sheep myoblast proliferation, which is consistent with its role in mouse muscle. There is a core promoter of the *HMGA2* gene located at NC_056056.1: 154134300-154134882 (-618 to -1200 bp upstream of the *HMGA2* TSS). The SNP rs428001129 within the core promoter significantly increases the promoter activity of *HMGA2* and is associated with sheep’s chest width and depth, providing candidate sites for marker-assisted breeding in sheep.

## Figures and Tables

**Table 1:** Sequences of primers and siRNAs.

ID	Sequence (5′-3′)	Product
siRNA-1	CCGGUGAGCCCUCUCCUAATT	\
	UAGGAGAGGGCUCACCGGTT	
siRNA-2	GAGACAUCCUCACAAGAGUTT	\
	ACUCUUGUGAGGAUGUCUCTT	
siRNA-3	GCCAGCAUUCAAUUUCUACTT	\
	GUAGAAAUUGAAUGCUGGCTT	
siRNA-NC	UUCUCCGAACGUGUCACGUTT	\
	ACGUGACACGUUCGGAGAATT	
CDK2-F	TGGGCCAGGCAGGATTTTAG	166 bp
CDK2-R	GTCGAAGGTGAGGTACTGGC	
CCND1-F	GCTTCCTCTCCTATCACCGC	149 bp
CCND1-R	GGCTTTGGGGTCCAAGTTCT	
GAPDH-F	GTCGGAGTGAACGGATTTGG	196 bp
GAPDH-R	CATTGATGACGAGATTCCCG	
HMGA2-F	AGACCCAAAGGCAGCAAAAAC	100 bp
HMGA2-R	GCCATTTCCTAGGTCTGCCTC	
P1	cgagctcttacgcgtgctagcAAAAGTTTTTATTTTGGAATTG	1942 bp
P2	cgagctcttacgcgtgctagcTCAGTGGAGGCTGGTGCG	1541 bp
P3	cgagctcttacgcgtgctagcCAGGTAAAGGCCAAGCCCC	1142 bp
P4	cgagctcttacgcgtgctagcGGATCCCCGCAGAATCTCC	560 bp
R	acttagatcgcagatctcgagTCGCCTCTGTCGCCCTGA	\
SNP-F	GCCTCCCTCCTCCTCATACT	492 bp
SNP-R	CGGCTTGGAAAGGGAAGAGA	

Note: lowercase bases are homology arm sequences matched to pGL3-Basic, and underlined bases are *Xho* I and *Nhe* I restriction sites.

**Table 2:** Association analysis between rs428001129 and sheep growth traits.

Age	Traits	Genotype			*p* Value
		GG	GT	TT	
birth	body weight	3.67 ± 0.81	3.73 ± 0.84	3.95 ± 0.94	0.250
	withers height	37.63 ± 3.13	37.90 ± 2.88	38.85 ± 2.47	0.141
	body length	30.46 ± 2.78	30.88 ± 3.01	31.17 ± 2.99	0.264
	chest girth	35.62 ± 2.89	35.82 ± 3.03	37.17 ± 4.54	0.055
	chest depth	15.60 ± 2.16	15.58 ± 1.52	15.90 ± 1.85	0.704
	chest width	10.16 ± 1.48 ^b^	10.44 ± 1.50 ^b^	11.06 ± 1.65 ^a^	0.009
	cannon circumference	5.83 ± 0.68	5.93 ± 0.70	6.06 ± 0.62	0.183
1 mth	body weight	10.22 ± 2.43	9.82 ± 2.24	10.30 ± 2.32	0.545
	withers height	45.74 ± 3.06	44.93 ± 3.11	43.85 ± 3.48	0.095
	body length	43.43 ± 3.83	42.71 ± 3.48	42.70 ± 2.91	0.455
	chest girth	50.06 ± 4.34	49.66 ± 3.65	50.30 ± 3.56	0.781
	chest depth	22.23 ± 1.86	21.95 ± 1.71	21.85 ± 1.62	0.568
	chest width	14.43 ± 1.51	14.24 ± 1.47	14.30 ± 1.67	0.725
	cannon circumference	6.50 ± 0.49	6.38 ± 0.45	6.45 ± 0.37	0.292
2 mth	body weight	17.85 ± 3.81	18.28 ± 3.89	18.64 ± 4.49	0.411
	withers height	49.75 ± 3.41	50.28 ± 3.58	50.68 ± 3.95	0.216
	body length	51.93 ± 4.54	52.53 ± 4.94	52.75 ± 4.81	0.409
	chest girth	59.85 ± 5.08	60.66 ± 4.82	60.96 ± 5.66	0.232
	chest depth	23.54 ± 2.22 ^b^	23.88 ± 2.19 ^ab^	24.61 ± 2.31 ^a^	0.040
	chest width	16.34 ± 2.23 ^b^	16.94 ± 2.40 ^a^	17.79 ± 2.94 ^a^	0.002
	cannon circumference	6.92 ± 0.58	7.00 ± 0.62	7.20 ± 0.79	0.062
3 mth	body weight	24.41 ± 3.95	24.44 ± 4.08	24.70 ± 3.90	0.981
	withers height	54.70 ± 3.02	54.87 ± 3.30	55.19 ± 3.27	0.912
	body length	56.64 ± 3.06	56.98 ± 3.24	58.06 ± 3.12	0.490
	chest girth	67.32 ± 4.27	67.13 ± 4.70	67.38 ± 3.42	0.973
	chest depth	26.83 ± 1.69	26.90 ± 1.70	26.81 ± 1.28	0.975
	chest width	17.71 ± 1.64	17.95 ± 1.53	18.13 ± 1.30	0.659
	cannon circumference	7.44 ± 0.44	7.36 ± 0.54	7.50 ± 0.38	0.632
6 mth	body weight	37.33 ± 7.39	37.50 ± 7.43	37.90 ± 8.65	0.848
	withers height	61.44 ± 4.25	61.83 ± 3.81	62.43 ± 4.26	0.292
	body length	66.18 ± 4.68	65.90 ± 4.62	67.33 ± 5.07	0.313
	chest girth	77.90 ± 6.00	77.70 ± 5.95	78.00 ± 7.40	0.969
	chest depth	28.66 ± 2.13 ^b^	28.82 ± 2.15 ^b^	29.72 ± 2.93 ^a^	0.047
	chest width	19.33 ± 2.21	19.47 ± 2.32	20.02 ± 2.30	0.269
	cannon circumference	8.05 ± 0.84	8.02 ± 0.81	8.38 ± 1.07	0.107

^a,b^ Values without letters or with the same letter in the same row indicate no significant differences (*p* > 0.05); values with different letters in the same row indicate significant differences (*p* < 0.05). mnt, month(s).

## Data Availability

All data are available within this paper.
